# Nanomedicine Faces Barriers

**DOI:** 10.3390/ph3113371

**Published:** 2010-10-28

**Authors:** Paul Debbage, Gudrun C. Thurner

**Affiliations:** 1 Department of Anatomy, Histology and Embryology, Innsbruck Medical University, Müllerstrasse 59, 6020 Innsbruck, Austria; 2 Department of Radiology, Innsbruck Medical University, Anichstrasse 35, 6020 Innsbruck, Austria

**Keywords:** nanomedicine, vascular barriers, glandular tissues, central nervous tissues, transbarrier targeting, lesion-specific targeting

## Abstract

Targeted nanoparticles have the potential to improve drug delivery efficiencies by more than two orders of magnitude, from the ~ 0.1% which is common today. Most pharmacologically agents on the market today are small drug molecules, which diffuse across the body’s blood-tissue barriers and distribute not only into the lesion, but into almost all organs. Drug actions in the non-lesion organs are an inescapable part of the drug delivery principle, causing “side-effects” which limit the maximally tolerable doses and result in inadequate therapy of many lesions. Nanoparticles only cross barriers by design, so side-effects are not built into their mode of operation. Delivery rates of almost 90% have been reported. This review examines the significance of these statements and checks how far they need qualification. What type of targeting is required? Is a single targeting sufficient? What new types of clinical challenge, such as immunogenicity, might attend the use of targeted nanoparticles?

## 1. Introduction

Nanomedicine offers the chance to transport hundreds of drug molecules to precisely the lesion they should modify, by use of small numbers of targeting groups. It thus holds out the hope of therapies in which all the target molecules in the lesion are saturated by the drug and the drug exerts no action anywhere outside the lesion, thus synthesizing insights due to Paracelsus, Ehrlich and Stumpf [1,2]. Nanomedicine is a new branch of medicine, barely 40 years old. Most of its promise remains to be realised, largely because humans have conceptual difficulties thinking at the micrometer scale of living tissues, and even greater difficulties at the nanoscale. This review thematises the opportunities and challenges of targeting in nanomedicine, and aims to provide a working guide to the unfamiliar micro- and nanoscale territories. Amongst the unfamiliar microscale phenomena are the complex blood-tissue barriers governing access to lesion sites.

## 2. The Targeting Challenge

In pathophysiological states cells express surface molecules which are absent in healthy states, or are strongly upregulated during the course of the disease. These molecules can serve as targets to which therapeutic agents can be directed, and in this review such cell surface molecules will be termed “target molecules”. Targeted particles should accumulate at the target molecules in the lesion, binding ideally to all of them, and no particles should remain outside the lesion. The drug they carry should bind to all the target molecules in the lesion and only to them, and the particles should release further drug molecules as target molecule recycling at the cell membrane makes new target molecules available. In this ideal case, neither excessive nor inadequate dosing occurs, so toxic side-effects on the one hand, and the failure to eliminate remnant malignant cells on the other hand, are both avoided. The matching of drug dose to target molecule site and number can result in major improvements in therapeutic efficiency [3,4,5,6,7,8]. The resulting “individual effective dose treatment” would be a significant step towards personalized medicine [9,10,11,12]. This ideal is not yet met in the complex multi-compartment organism that is the human patient. In a 75 kg human body a 1 gm tumour represents 0.001% of the body volume; passive distribution, accessing all points of the organism equally, would result in 0.001% of an active agent accessing the tumour and implies a pharmaceutical efficacy clearly below this value. Source and sink effects, such as the presence of influx pumps at target cell surfaces [13,14,15,16], increase this percentage, but in real organisms the improvement is limited because drugs distribute into many compartments within the organism. It has long been known that in the real clinical world, and even allowing for the facilitation provided by enhanced permeability and retention (see below), many directed therapeutic agents fail to reach their target cells [17,18,19,20,21]. Only a small proportion of intravenously applied monoclonal antibodies - which are targeted nanoparticles in the 10 nm size range - reach their targets on parenchymal cells [22,23,24]. Accumulation of targeted peptides and of monoclonal antibodies with 0.2-0.4% uptake is slow and can require up to a week [17,25,26,27,28,29,30,31]: the uptake efficiency can be as low as 0.01% [32] or less [33]. The reason for the low uptake efficiency lies primarily in the fact that, during most of their stay in the bloodstream, the antibody molecules remain further than 1 meter distant from the target molecules, whereas specific binding only occurs within a distance less than 100 nanometers - that is, ten million times closer (see below). It is evident that targeting requires considerably more finesse than simply providing a particle with a targeting moiety aimed at the final biomarker molecule characteristic of the lesion cells. Nonetheless, numerous monoclonal antibodies which fit this description are presently in clinical trials or just entering clinical application, and represent commercial markets worth many billions of dollars [http://www.path.cam.ac.uk/~mrc7/humanisation/antibodies.html, http://www.gallartinternet.com/mai]; [34,35], so that targeting efficiencies near 1% represent the state of the pharmaceutical and clinical art today.

### 2.1. Clinically Relevant Targets

Haematological malignancies can be easily targeted, because the lesion consists of cells within the bloodstream. There are no physical barriers between the blood-borne nanoparticles and the lesion cells, so that only one type of molecule must be “recognised” as a target by the nanoparticle. Lesions located at one of the body surfaces, such as the skin or one of the several mucosae (urogenital, intestinal, buccal/nasal), can also be directly accessed, although if they are malignant tumours their metastases are generally located far from body surfaces; examples include endometrial and urothelial carcinomas, the cancers of the airways and the digestive tract, and melanomas. However, most oncological entities are solid tumours lying deep within one of the body’s organs; frequently the organ is a gland. If a tumour-specific, validated and prognostically relevant biomarker molecule has been identified for one of these tumours, then this molecule will be present in the tumour and ideally on the surface of the tumour cells, and small amounts of it may also be detectable in the bloodstream; the fraction detectable in the bloodstream is useful for detection and monitoring of the malignancy, but not for targeting purposes. Similar considerations apply for all diseases which run major parts of their course behind the protection of a blood-tissue barrier (see below). To stay with the example of a carcinoma, a clinically relevant target will be a tumour-specific, validated and prognostically significant molecule or part of a molecule, located on the surface of the carcinoma cells, probably hiding deep within a glandular tissue behind a blood-tissue barrier. One familiar example is the prostate membrane specificantigen [36,37,38], and this corresponds to the single target that must be recognised to access a cell within the bloodstream. In order to reach this “final” target molecule however, a nanoparticle navigating the multistage path through the blood-tissue barriers of the body must first recognise at least one other type of target molecule in addition, as described below. In fact, for each additional barrier that must be navigated a further separate, independent target molecule must be recognised.

### 2.2. The Multistage Path to the Target

**Figure 1:**
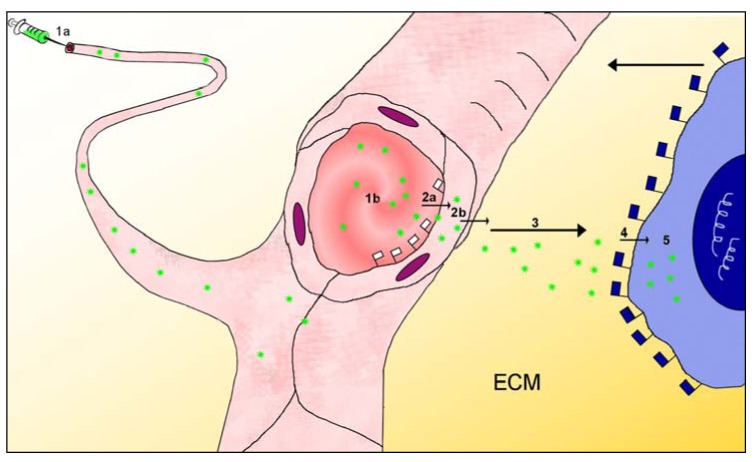
This sketches the first four stages of the multistage targeting path which particles (green stars) must navigate after injection (syringe, 1a) into the bloodstream (red blood in pink blood vessels), in order to access a lesion-characteristic cell such as a tumour cell (blue cytoplasm, dark blue nucleus). The injection site, top left, may be more than a meter distant from the lesion; this sketch ignores the differences between large and small blood vessels. *Stage 1* begins at the injection site (1a) and ends at the patch of endothelium (1b) in a microvessel overlying the lesion; the microvessel is shown sectioned at this site. Lesion-flagging molecules embedded in the apical surface of the endothelial cells at this site (and nowhere else) (white flags) indicate that a lesion is hidden behind the microvessel wall at this site; at this point the particles leave the bloodstream (*stage 2a*), cross the endothelial cell forming the microvessel wall (2b) and enter the sub-endothelial interstitial compartment (“perivascular space”). *Stages 2a and 2b* together require the particle to pass across the endothelium, which is the usual site for the blood-tissue barrier; the distance across the endothelial wall is approximately 0.000002 meters = 2 µm. *Stage 3* of the multistage path involves crossing the interstitium (3), which contains several types of obstruction as shown in Figure 2. *Stage 4* involves the binding of the particles to the target molecules on the surface of the lesion-specific target cell, and this binding is also mediated by flag molecules (blue flags) indicating that the cell is a lesion-specific cell. This review does not follow the nanoparticles into the target cell (*Stage 5*) (for this, see Torchilin [40]).

Since most drugs relevant to oncology are applied via the bloodstream, this review will focus mainly on the intravascular application of targeted drugs, especially in the form of nanoparticulate formulations. The path from bloodstream to the target molecule, which is located within a lesion deep within an organ, consists of a series of stages: each stage excludes a fraction of the particles from further targeting progress, and thus reduces overall targeting efficiency. Most organs maintain internal compartments, with physical and functional barriers separating their internal milieu from the bloodstream. The first requirement of the targeting task in nanomedicine is therefore to analyse the path the particles must navigate from the blood to the immediate vicinity of the biomarker molecule in the target tissue. Previous authors have already described aspects of the multistage path from injection site to tumour [23,39]. This review distinguishes five stages of the path to the target molecule (Figure 1), the first beginning at the intravascular injection site and ending at the apical surface of the endothelial cells overlying the lesion, the second being passage across the vascular wall to enter the perivascular space within the lesion, the third being movement across the interstitium in the perivascular space towards the close vicinity of the target molecules, the fourth being the movement across the last50-100 nanometers during which the targeted particle adheres specifically to its target molecule on the lesion-specific cell, and the fifth being the entry into the target cell and execution of actions to incapacitate or destroy that cell. This review focuses on the design of nanoparticles which should access the target molecules on the cell surfaces of the lesion; the review therefore accompanies the particles to the end of *stage 4*.

*Stage 1: Injection site to target endothelium.* *Stage 1* of the multistage targeting path involves passage through the bloodstream, starting from the site of injection (intravenous or via an implanted port, more rarely intra-arterially). This route is often a large fraction of one meter in length and the injected material passes through the large-diameter vessels of the systemic circulation, prior to reaching microvessels near and within the lesion. The bloodstream, turbulent in many places, mixes intravascularly applied materials rapidly. After a few passes through the circulation - that is, after at latest one or two minutes - substances introduced at one site into the blood can be considered sufficiently mixed to be homogenously distributed throughout the blood compartment [41]; this review will therefore ignore local blood concentrations of the particles, and will consider the targeting of nanoparticles which are located simply “in the blood”. After injection, the nanoparticles traverse the bloodstream and within one minute they encounter the patch of endothelial cell wall overlying the target lesion. This is the “target area of endothelium”. It hides a target lesion such as a solid tumour, and it is small compared to the total area of endothelium within the body, for example: in a person weighing 70 kg, a 1 cm^3^ solid tumour (50% hypervascularised) has a vascular bed with an endothelial apical surface which comprises approximately 0.002% of the entire endothelial surface in that person. When nanoparticles are injected into the vascular system, their first pass across the target area of endothelium is likely to saturate any relevant target molecules located there, which will bind (and thus remove) a fraction of the targeted nanoparticles. Several further passes of the particles across this target area of endothelium will take place during the next 2-3 minutes, and will usually occur before the relevant target molecules appear again at the endothelial apical surface: the recycling rates for target molecules are close to 0.05 min^−1^ [42] implying that the full complement of target molecules will return to the cell's surface only after15-20 minutes. As comparison: for a potential target molecule, the folate receptor on tumour cells, a recycling time of 8 hours has been measured [43]. During each pass, the specific target-site sink for the nanoparticles is generally far smaller than the supply of nanoparticles in the blood. Moreover, other sinks exist. One of these is the vascular bed of the liver, which in a 70 kg human body weighs 1.2-1.5 kg and comprises approximately 2% of the entire vascular bed: it is therefore about 1,000 times larger than the target endothelial surface in a 1 cm^3^ solid tumour; the hepatic vascular bed is also densely occupied by macrophages (von Kupffer cells), which may phagocytose the nanoparticles at a faster rate than the target vascular bed can transport them into the tumour. Amongst other sinks, the reticulo-endothelial system is important, taking up nanoparticles and accumulating them in lymphatic tissues [44]. Further sinks include any sites of inflammation present in the body, and there are yet other sinks within the kidney. Due to the presence of multiple sinks, the bloodstream will eventually be entirely depleted of the particles: at this time any particles remaining in non-target tissues have failed to reach their targets and this failure reduces overall targeting efficiency. The above discussion began with a bolus injection, but similar considerations apply for the case of a continuous infusion of particles.

*Stage 2: Target endothelium to lesion interstitium.* The nanoparticles arriving at the target area of endothelium overlying the lesion will usually be confronted by structural and functional barriers which exclude particles from the internal compartment of the tissue (Figure 1), thus preventing them from accessing the lesion containing the target biomarker molecules. To access the lesion, they must cross the endothelial barrier (if the lesion has rendered the vascular wall permeable, then the barrier will be reduced or absent and the particles may accumulate in the underlying perivascular space due to “enhanced permeability and retention” (EPR), see below). Particles failing to cross the barrier at the first pass will remain in the bloodstream, and may cross the barrier at later passes through the circulation. The blood-tissue barrier, noted here as *stage 2* of the multistage targeting path, is a major challenge in nanomedicine because it obstructs the passage of the great majority of targeted nanoparticles. They fail to cross this barrier at each pass, remain in the bloodstream, and are eventually taken up into the other sinks mentioned above. It hinders access for essentially all lesion-specific monoclonal antibodies [17,31,33,45,46], and is the main explanation for their low targeting efficiency *in vivo*, noted above. A mechanism allowing particles to cross this barrier efficiently is one of the major requirements for achieving “personalized medicine” today. This review therefore returns to these blood-tissue barriers as a major topic, see Section 2C below. Blood-tissue barriers are an unfamiliar concept because they only function in the living organism: tissue sections as prepared in pathohistology cut such barriers open, providing unhindered access to antibodies applied in immunohistochemistry; in cell cultures barriers are only present if considerable ingenuity was used to establish them there, and in cell suspensions (used in most drug-screening procedures) they are lacking entirely. In striking contrast, they are ubiquitous in the living organism.

**Figure 2:**
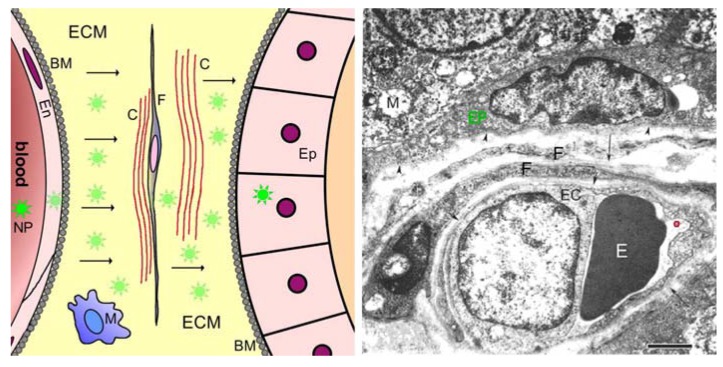
Barriers between blood and glandular epithelium, sketch at left, electron micrograph at right. The image at left is a sketch diagram of structural barriers between blood and parenchymal epithelial cells (Ep) of an organ (e.g., gland). This Figure shows details omitted in *stage 3* of Figure 1. After crossing the blood-tissue barrier (*stage 2*) consisting of the endothelial cell sheet (En), the subendothelial basement membrane (BM), pericytes and (possibly) smooth muscle cells, the nanoparticles (NP, green stars) must cross (arrows) the perivascular compartment, which contains the extracellular matrix (ECM) consisting of glycosaminoglycans, proteoglycans and proteins, the collagen fibrils and fibril bundles (C), the sheetlike processes of fibroblasts (F), occasional nerve fibres and immune cells such as macrophages (M), and the subepithelial basement membrane (BM). The image at right is an electron micrograph showing these barriers in a tissue specimen. The image shows a capillary in the peritumoral region of a prostate carcinoma of human (from Wieser *et al.* [51]). In this peritumoral region, the blood-tissue barrier is essentially intact (truly normal tissue of human prostate gland is difficult to obtain in a good state of ultrastructural preservation!). The arrowheads and arrows all point to basement membranes. Nanoparticles in the blood volume (a rather large one is encircled red) can interact with cells in the blood (such as the erythrocyte, E) or with the apical surface of the endothelial cell (EC). Lying beneath the endothelial cell layer are the subendothelial basement membrane, then the extracellular matrix containing collagen fibres and glycosaminoglycans. Within the extracellular matrix are embedded fibroblasts (F), two layers in this case, then resting upon its own basement membrane the prostate glandular epithelium (EP, green text), which is the target. The washed-out state of the mitochondria (M) in the epithelial cells is due to the period elapsing between operative removal of the tissue and fixation in aldehydes. Calibration bar: 2 µm.

*Stage 3: Lesion interstitium to neighbourhood of target molecule*. Targeting *stage 3* begins at the basal (abluminal) surface of the endothelial cell layer, which in the case of microvessels lies directly within the perivascular space; larger vessels are surrounded by more or less elaborate layers of smooth muscle cells, forming a significant obstruction between the endothelial lining of the vessel, and the interstitium. Starting from the immediate environs of the blood vessel within the lesion, the particles must now “migrate” to the close vicinity of the target molecules. The path within the interstitium is short, usually only several micrometers long. This path was reviewed in detail byJain [23]. It is obstructed by the structures shown in Figure 2. These include the various types of cells present in the connective tissue in this compartment (fibroblasts, fat cells, plasma cells, macrophages), and also the various types of fibres and fibrils that are present there, including collagen and elastic fibers. Not only do these all form stable structural barriers which must be circumvented, but the glycosaminoglycan and peptidoglycan molecules forming the basic structural elements of the “extracellular matrix”, which bear charges and which each spatially organises hundreds of water molecules, also exert dragging forces slowing the movement of the particles. Amongst these fibres and sheetlike cell extensions the nanoparticles will be scattered and slowed down. There are however no fully enclosed spaces here, so the major effect of the obstructions is to increase the time required for the particles to reach the close vicinity of their targets. In addition to the physical barriers presented by the bulk of the cells and the fibres, particles may adhere to the cells and fibres, due to undesired chemical affinities or to charge effects. At the point closest to the target cells in the lesion, the particles may encounter an intact, or partially intact, basal membrane, which forms a charge- and space-filter between target epithelial cells and the extracellular matrix [47,48,49,50].

*Stage 4: The close vicinity of the target molecule.* The fourth stage of multistage targeting, occurring at the end of a meter-long targeting pathway and commonly but erroneously considered to be the single requirement for overall targeting, occurs within the final micrometer before the targeted molecules. Consider an antibody molecule as a targeted nanoparticle: its stereospecific binding affinity only becomes effective at a distance less than 100 nm from the target [52]. This is also true for other targeting groups such as lectins, aptamers or enzymes. This final adhesion of the targeted nanoparticle to its target molecule is the step which is generally termed “targeting”, ignoring for example the prior targeting stage necessary to cross a blood-tissue barrier. As the targeted nanoparticles finally come within the nanometer range allowing stereospecific binding, they encounter an array of the specific target molecules (in the sense of this review), which ideally are embedded in the plasma membrane at the surface of the lesion-specific target cell. Allowing one targeting spike per 1,000 nm^2^ on the nanoparticle (see below), then [1 µm^2^/1,000 nm^2^] 1,000 target molecules per µm^2^ on the cell surface offer a one-to-one binding opportunity for the targeting spikes. Atomic force microscopy does indeed visualize approximately 1,000 protein molecules/µm² at the plasma membrane surface [53]; it follows that molecules should be selected for use as biomarker targeting molecules which are expressed at a minimum density of 1,000 molecules/µm² at the target cell surface.

This binding step, which attaches the particle to a target cell, can be mediated by antibodies which are presently commercially available. For example, the anti-Epithelial Membrane Antigen (anti-EMA) binds to different types of secretory (glandular) epithelia. Anti-prostate-specific membrane antigen (anti-PSMA) antibody targets the prostate gland [36,37,38], anti-thyrotropin receptor antibody targets the thyroid gland [54,55], anti-prolactin receptor antibody targets the mammary gland [56], and Adrenal Cortex Inner Zone Antibody (IZAb) targets the adrenal gland [57].

*Stage 5: Entry into target cell.* In many cases, the drug contained within the nanoparticle is designed to interact with structures inside the target cell. The cell membrane, and behind that the plethora of intracellular compartments, form a further barrier at the fifth stage of the targeting path. It may be sufficient for the nanoparticle to release its drug load into the cell by injecting it through the cell membrane into the cytoplasm, and this is the procedure followed by natural infectious nanoparticles such as the T viruses [58]. For the case that the entire nanoparticle should enter the cytoplasm, a wide range of strategies has been evolved, well reviewed recently by Torchilin [40].

### 2.3. Barriers between Blood and Tissues

As described above in section 2.2, *stage 2* of the multistage targeting path generally involves a barrier which separates the blood milieu from the specific milieu required for optimal functioning of a particular tissue. Since each tissue has one or several physiological functions specific to itself, a range of different barriers is necessary to protect the numerous specific internal environments of the various tissues within the body. The blood environment contains many materials which are unnecessary or harmful in the context of specific physiological functions, and the barriers are therefore adapted to regulate *flows of materials* between the blood and the tissue internal compartments. In addition, tissues are protected against the *unregulated ingress of cells*, which may include the body’s own cells such as those of the immune system, but also alien cells such as bacteria, fungal cells, *etc.* In sum, in most organs, blood-tissue barriers regulate the passage of cells and materials between the blood compartment and the tissue parenchymal compartment, thus providing controlled environments for tissue-specific function. The terminology “barrier” suggests a simplicity and uniformity of structure and function, but in reality blood-tissue barriers are not only complex (Figure 2), they are also extremely varied. They consist partly of continuous sheets of endothelial or epithelial cells joined by tight junctions with varying degrees of closure [59] but partly also of subendothelial basement membranes forming spatial and charge barriers [47,48,49,50]. In addition, enzymes and charged molecules present at the endothelial surfaces form additional specific barriers to certain classes of molecules [60,61,62,63,64]. Blood-tissue barriers are generally under regulatory control [65,66,67,68,69]. The functions of blood-tissue barriers include the maintenance of controlled ionic environments essential for nerve impulse transmission [70], protection against infection [71,72,73], regulation of immune cell access to secretory products [74], regulation of secretion by exocrine glands [75,76] or of hormone passage into the blood from endocrine glands [77]. Blood-tissue barriers utilise a range of structures and mechanisms to regulate cell and material ingress, and it is likely that there are as many types of barriers as there are types of tissue. During the last 100 years numerous organs were examined for the presence of barriers, and some barriers have been well characterised, though none is completely understood. The complexities of the numerous tissue barriers means that evidence for blood-tissue barriers can be of several types. For example, the presence of a barrier to proteins necessitates the additional presence of mechanisms to facilitate passage of particular desired proteins; the presence of specific protein transporters thus provides evidence for a barrier to proteins in general. Similarly, the exclusion of molecules from a tissue compartment, the presence of differential transport rates for certain molecules, the presence of barrier-forming enzymes, or a demonstration of morphological evidence for tight junctions or intracellular transport vesicles, are all accepted as evidence that a blood-tissue barrier is present within an organ. A large number of studies were carried out in the 1960s-1970s, revealing a range of barriers in different tissue types [78,79], and showing that the blood-tissue barriers in different organs vary widely. The liver, for example, has no barriers, whereas the kidney contains a full spectrum of them, ranging from low to very high barriers. This review considers two blood-tissue barriers in more detail: the blood-brain barrier and theblood-milk barrier.

#### 2.3.1. Blood-brain barrier

**Figure 3:**
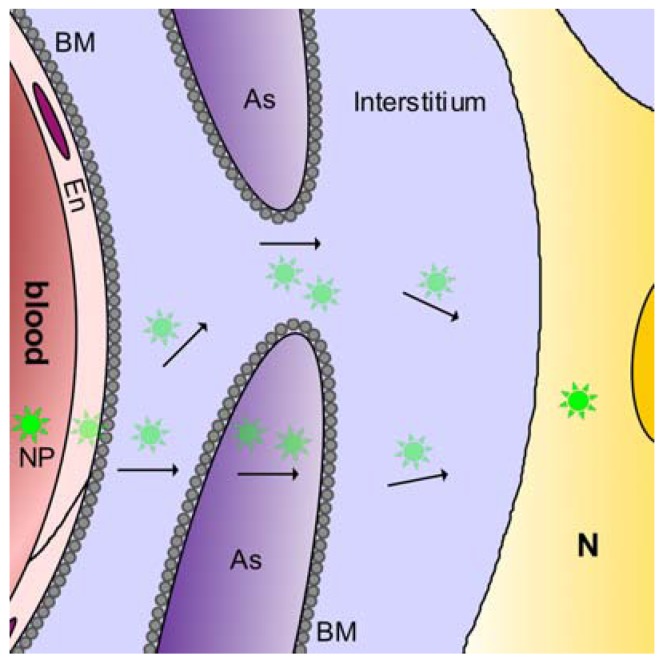
Shows possible transbarrier transport paths for nanoparticles across the barriers between the blood and the central nervous tissue. Nanoparticles (NP, green stars) applied in the blood could cross the endothelial layer (En) and the subendothelial basement membrane (BM), and pass through the gaps between two astroglial endfeet (interstitium). Alternatively, they could be transcytosed across the astroglial endfeet (As), to reach the central neurons (N).

The “blood-brain barrier” of the central nervous tissues was the first blood-tissue barrier to be recognised, in the late 19^th^ century [80,81,82,83]. It was studied extensively after the 1960s, when its morphological basis was recognized to be the endothelial layer of the cerebral microvessels [84,85]. The tight junctions between the microvessel endothelial cells exhibit high degrees of closure, effectively blocking the paracellular pathway. Any materials in aqueous solution must therefore cross the endothelial layer by transcytosis, which is strictly regulated in central nervous tissues [67,86]. This is the “tightest” or the “highest” of all the vascular barriers. The microvessels are closely enwrapped by astroglial endfeet processes (Figure 3), which form an almost closed sheath around the microvessel [87]. Thus although the endothelial cells form the blood-brain barrier, any materials passing the endothelial cells to enter the brain interstitium will enter a narrow space between the endothelial cells and the astroglial endfeet. Exit from this space is either by passage across the glial endfeet processes, or through the gap between adjacent endfeet (Figure 3), to enter the wider interstitial compartment of the central nervous tissue. The neurons and their axonal and dendritic fibres densely fill this compartment; they are generally also ensheathed by astroglial and oligodendroglial wrappings. Movement in this densely filled compartment is restricted by these numerous structures. Materials in this interstitial compartment can enter cell membranes or even enter neuronal cells by uptake at synapses between the nerve cells and fibres (see below). Detailed knowledge about the blood-brain barrier has been obtained by microscopy, including immunohistochemistry [88], by electrophysiological studies [89], by biochemical studies of the barrier components [90], by tissue culture modelling [91] and by knockout studies of essential barrier components [92]. The scientific and clinical literature encompassing studies on the blood-brain barrier contains thousands of relevant manuscripts, and frequent and regular reviews are written about this barrier [93,94,95,96,97,98,99,100]. We note briefly here the existence of the two other “high” barriers: the blood-testis barrier [101,102], and the blood-placenta barrier [73,103,104,105,106,107,108]. It is interesting to compare the strikingly different structural embodiments of these three “high” barriers: the blood-brain barrier resides in the microvascular endothelium of the cerebral circulation, the blood-testis barrier resides in the Sertoli (epithelial) cells of the testis, and the blood-placenta barrier resides in the syncytial trophoblast. Each of these barriers therefore bases on a different morphological substrate: an endothelium, an epithelium, and a syncytium. This illustrates the great variety ofblood-tissue barrier constructions that are possible, and a wide variety of constructions is also likely to be found amongst the “lower” or “less high” barriers found in other organs.

#### 2.3.2. Blood-milk barrier

Blood-borne agents do not generally have access to the parenchymal tissue cells of glands, which are separated from the blood milieu by barriers which are both functional and significant [75,109] (Figure 2). This review considers the blood-tissue barrier in one glandular tissue, the mammary gland. Mature milk is a complex mixture of membrane-bound fat droplets, casein micelles, and an aqueous phase containing lactose and complex carbohydrates, minerals, proteins and other soluble components [110]. This mixture arises from the secretions produced in different secretory pathways, sometimes in different epithelial cells and sometimes in the same cells; a complex of secretory routes is involved, including the membrane pathway, the Golgi pathway, the milk fat pathway, the transcytotic pathway and the paracellular pathway [110,111,112]. Secretion utilising these pathways is influenced by the functional state of the gland, and by hormones and growth factors [111]. The milk compartment contains milk proteins and lactose, and low concentrations of sodium and chloride, whereas the interstitial fluid contains plasma proteins and high concentrations of sodium and chloride. The blood-milk barrier separates these two compartments and regulates the milk compartment. The structure and function of the murine blood-milk barrier was described in detail by Monks and Neville [113], building on data from earlier authors to analyse specific transport mechanisms in the lactating mammary gland, and highlighting the complexity of the blood-milk barrier. It resides largely in the impermeable glandularepithelium [112,114,115,116], though material flows across the capillary endothelium are also regulated. In addition to the several secretory pathways mentioned above, the blood-milk barrier contains several different transcellular transport systems for proteins, including transferrin, immunoglobulins and albumin. We consider albumin transport in more detail. Three transport systems exist for albumin alone, as it crosses the capillary endothelium and the glandular epithelium; a balance of albumin concentrations arises in the interstitium as a result of albumin uptake into the milk, its uptake out of the blood and its recycling into the blood plasma. Albumin passage into the milk, across the lactating epithelium, occurs only in the basolateral to apical direction [113,117], and is independent of both transferrin transport and of the protein secretory pathways. In the blood-to-milk direction albumin is transported in two different systems sequentially, namely in caveolae across the capillary endothelium, but in clathrin-coated vesicles across the lactating epithelium. The recently emerging picture is that the blood-milk barrier is strictly regulated and is complex both morphologically and physiologically, involving multiple cell layers and also having multiple specific protein transporters functioning not only in both directions (blood-to-milk and milk-to-blood) but also independently of one another (and utilising transport systems of great relevance for the transport of nanoparticles). Some parts of the complex machinery of this barrier are specific to the mouse, which passes unusually large amounts of albumin into the milk (20 mg/mL) [113]. Rats pass little albumin into the milk (5 mg/mL) [118], cows less(0.4 mg/mL) [119] and humans also little (0.4 mg/mL) [120]. The elaborate barrier structures characterising the murine blood-milk barrier may therefore be present in reduced form or in quite different form in ruminants and humans. A detailed analysis of the blood-milk barrier in humans, with its ethical implications on the one hand, coupled with its importance for the nanomedical therapy of breast cancer on the other hand (see below), remains to be undertaken and is one of the crucial topics on the agenda of nanomedicine, as explained below. The above discussion of material movements between blood and milk has ignored the movement of cells, for example of the immune cells (neutrophils) which are the primary defence of the gland against extravascular infection [121], and therefore presents a simplified view of this barrier. Movement of cells across the blood-milk barrier only occurs if signals from the site of infection (the interstitium) pass from the interstitium to the luminal side of the microvascular endothelium in order to flag the site as a target endothelium for the neutrophils [122], and these cells must then navigate the vascular wall structures in order to migrate from the blood to the site of infection in the interstitium. The complexity of cell migration through the milk gland barrier has led researchers to create models in order to understand the diapedesis of the neutrophils through the vascular wall structures [123]. In summary, the blood-milk barrier, which regulates movement both of cells and of many different types of materials, is of critical importance in nanomedicine, not only in relation to its important role in the therapy of mammary carcinomas (see below), but also as an example of the barrier complexity which will be encountered as a routine challenge in targeting nanoparticles to essentially any of the tissues of the body.

#### 2.3.3. Blood tissue barriers in muscles

In both cardiac and skeletal capillaries, the endothelial lining is permeable to plasma proteins and to colloidal particles [48,124,125,126,127]. Both vesicular transport and paracellular diffusion are involved [128,129,130,131,132], though assigning their relative importance offered difficulty [133,134]. In contrast, the microvessels in the smooth muscle of the ciliary muscle of the eye maintain a tight barrier [135], as do those of the uterus [68]. The example of the three different types of muscle (skeletal, cardiac, smooth) shows that the vascular-tissue barriers in muscles do not function in the context of the intracellular machinery which equips the muscle cells for contraction, but instead are adapted to the functions which the muscle tissues carry out within the different organs of which they form an integral part.

#### 2.3.4. Blood-tissue barriers in other organs

Blood-tissue barriers have been documented and investigated in most tissue types, the following is a brief list excerpted from the voluminous literature. In lung, a less permeable barrier is maintained than in muscle [136,137,138,139,140]; barriers of different “tightness” or “height” are documented in the organs of the immune system [141,142,143,144], in the gut [145,146], in the hepatobiliary system [147], in bone [148] and in the skin [149], amongst others.

### 2.4. Blood-Tissue Barriers in Disease States, EPR

Lesions often exhibit significant vascular abnormalities, including hypervascularity, enhanced vascular permeability due to production of vascular permeability factors, and absence of lymphatic drainage [150]. These abnormalities result in partial or total failure of the blood-tissue barrier functions, as documented for example in the brain [151], in the mammary gland [121,152], and in non-malignant lesions and in malignant tumours of a wide range of tissues [153,154,155]. The enhanced permeability can be used as an approximate prediction of tumour grade in brain tumours [156] and is associated with malignity in experimental mammary carcinomas [157]. In addition, the neo-angiogenesis associated with rapid tumour growth [158,159] produces microvessels which are morphologically [160] and physiologically [161] abnormal and do not maintain mature blood-tissue barriers [162], and this can also be visualised in experimental mammary carcinomas [163,164]. At such permeable sites, large molecules and nanoparticles “wash in” to the perivascular space within the lesion, and since they are not “washed out” they accumulate there; this is known as the “enhanced permeability and retention effect” (EPR effect). This concept was coined in the oncological setting, where it has been extensively reviewed [23,165,166,167,168]. Raised vascular permeability is however a feature of inflammation in general and EPR may provide a useful clinical approach in non-oncological inflammatory conditions, such as colitis [169] or rheumatoid arthritis [170,171], myocardial infarct [172], or multiple sclerosis [173,174]. EPR thus provides a clinically useful opportunity to achieve raised concentrations of large molecules and particles in the interstitium of many lesions, but since multiple sites of raised vascular permeability may be present in a single patient and only some of these relate to the target disease, EPR is not a true form of targeting.

### 2.5. Lesions behind Barriers

**Figure 4:**
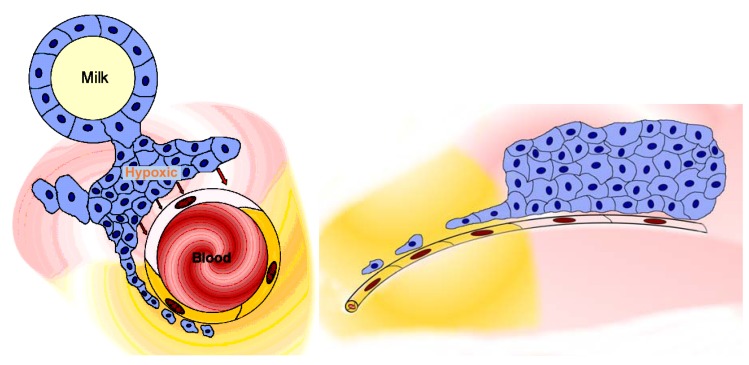
The tumour reduces or removes the blood-tissue barrier. Image at left: the sketch shows a microvessel (containing blood) within a tumour, a mammary carcinoma (an alveolus containing milk is shown to represent the glandular tissue). The tumour mass (blue cells) is hypoxic and releases factors (such as HIF-α, hypoxia-inducible factor α) whose action renders the endothelial cell layer of the microvessel permeable (pale pink endothelial cell); the permeable microvessel allows blood-milieu components entry to the interstitium around the tumour (moderate pink). A narrow extension of the tumour consists of a strand of tumour cells lying close to the microvessel and therefore well-oxygenated; these cells produce no HIF-α, so the nearby endothelial cell layer (yellow cells) remains “tight” and maintains the blood-gland tissue barrier, and the interstitium remains free of haematogenous elements (yellow). Image at right: the sketch shows a lateral view of the situation shown in the image at left, and demonstrates how the outlying strand of tumour cells can extend a considerable distance from the tumour and lie deep in the barrier-protected interstitium. These are the (very few) cells which will survive chemotherapy with conventional small-molecule drugs, and later generate filiae, resulting in the relatively high mortality due to metastasis that is associated with breast cancer. These are the cells which must be accessed via transbarrier targeting, see Figure 6.

Although disease states often render blood-tissue barriers permeable, many clinically refractory lesions reside behind intact and significant vascular barriers. Important diseases of the CNS, such as most stages of both Alzheimer's disease and Parkinson's disease [175,176] reside behind the blood-brain barrier. Although, in general, malignant tumours are associated with blood-tissue barrier breakdown, this is only true at advanced stages of solid tumour growth. Until tumours reach a certain degree of malignancy, and a certain size, they do not disturb nearby intact vascular barriers [177,178]. Furthermore, even tumours with generalised breakdown of the blood-tissue barriers, such as large tumours, have important regions, for example the growing/migrating leading edges of the tumour mass, which reside behind intact vascular barriers (Figure 4). The early development of a carcinoma is a further example: the malignant transformation of the gland epithelial cells involves several early stages which are hidden behind intact blood-tissue barriers. Micrometastases are comparable to early stages, in that they remain hidden behind intact vascular barriers [177]. These biological facts entail significant clinical consequences and make the nature of the vascular barriers a matter of great clinical importance. It would be of considerable value to study the blood-tissue barriers located within the different types of glands, because these barriers hide the earliest cellular stages of several of the most important types of carcinoma. At present, early-stage tumours, tumour leading edges, and micrometastases, all remain undetectable and inaccessible to diagnosis or therapy, especially by use of nanoparticles [179,180], and see below. It is in the advanced stages of disease that the blood-tissue barriers open, yet the early stages of malignant lesions, consisting of small numbers of cells, are in principle more amenable to therapy than the well-established lesions. Behind the barrier there are only a few cells in a micrometastasis or extending out from a tumour edge, but these are cells which will seed further filiae of the tumour and, finally, be responsible for the high mortality rates of malignant disease.

## 3. Particles Face Barriers

### 3.1. Small Drugs Pass Barriers by Diffusion

Under certain conditions, small drug molecules can traverse blood-tissue barriers by passive diffusion: if the molecules are small in size (<400-600 Da) [181], exhibit a certain degree of lipophilia and are present at least partly in non-ionised form, then they partition between the aqueous and lipid phases present within tissues. By varying the charge on the molecules, the nature of the derivative groups attached to them, and their lipophilicity, their passage through lipid membranes of the endothelial and epithelial cells, and thus through blood-tissue barriers, can be achieved. During this passage, a concentration gradient arises across the cell membrane, so that raising the blood concentration of the drug increases the total mass of drug molecules that accesses the lesion (http://www.merckvetmanual.com/mvm/htm/bc/190102.htm). The presence of intact blood-tissue barriers therefore hinders but does not prevent the therapeutic action of small molecules. Since the molecules enter the parenchyma of all tissues, though at different rates, they will function as active agents in all organs accessible by passive diffusion along a concentration gradient. They access essentially the entire volume of the body, so their therapeutic efficiency is limited to less than 1%, as noted above. The morbidity caused by the large fraction of drug molecules which does not access the lesion, but instead accesses other organ systems, the “side-effects”, sets an upper limit to further dose increases: the therapeutic efficiency of the drug is “dose-limited”. Beyond a certain point, doses of small-molecule drugs cannot be increased any further due to their lethal action on the patient, so that, for example, the dose which can be delivered to a tumour is inadequate to destroy all the tumour cells [182]. The side-effects of dose-limited agents limit the maximum tolerable dose and ultimately lead to inadequate therapy of the lesion. The resulting balancing act, aiming to find an optimum dose between efficacy and toxicity, particularly in drugs with narrow therapeutic index such as those commonly used in oncology, occasions much discussion at present [183,184].

### 3.2. Differentiated Delivery Strategies Optimise the Use of Nanoparticles

**Figure 5:**
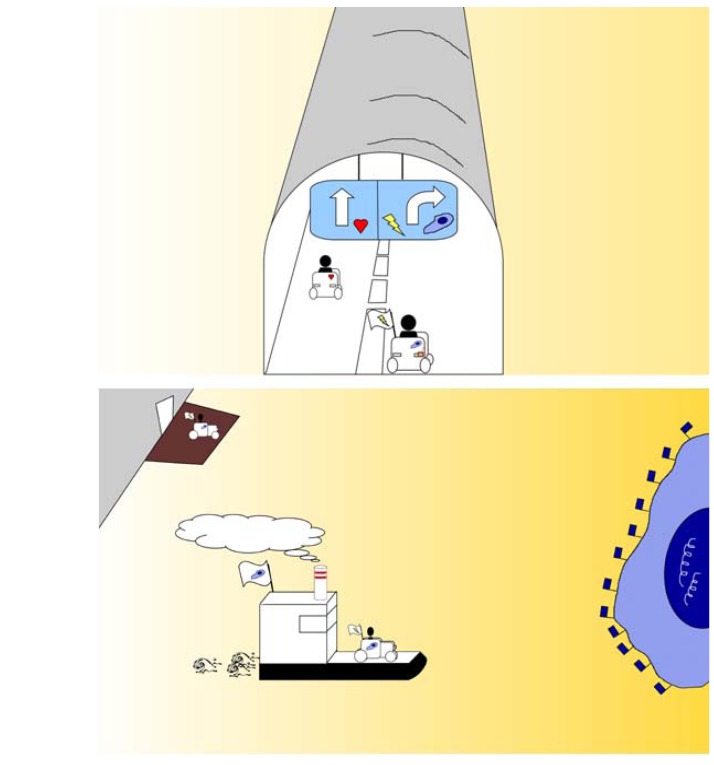
These two sketches thematise “flagging and ferrying”. The upper image represents the blood microvessel as a tunnel, such as found along a motorway. The vehicle at left (representing a nanoparticle) encounters no road-sign or flag to show the route to a lesion, and therefore continues driving straight. The vehicle at right encounters a roadsign or flag showing that a lesion can be found behind the tunnel (microvessel) wall and that a right turn will access the lesion. The lower image shows the vehicle after turning right out of the tunnel (microvessel) and now packed onto a ferry (for example, a caveolus) which is transporting it to the lesion, a tumour cell, shown as a blue island bearing blue flags at the right side of the image.

Nanoparticles, typically 30 nm or more in diameter, are too large to pass between the phospholipid molecules forming the cell membrane, and also too large to pass through the paracellular route which is limited by the width of the tight junction cleft. Thus, whereas small drug molecules cross blood-tissue barriers slowly, they do cross them: nanoparticles cannot cross them at all. This is a fundamental difference and underlies the high efficacy and selectivity which can be achieved by using nanoparticles. Intact blood-tissue barriers exclude non-targeted macromolecules and nanoparticles from the perivascular compartment [185,186]. Without special preparation, nanoparticles do not enter organs which maintain blood-tissue barriers, and cannot deliver drugs to these organs. Increasing the nanoparticle concentration does not enhance penetration through the barrier, because no concentration gradient arises across the barrier. Since the drugs cannot access non-target tissues, they can cause no “side-effects” there. These facts distinguish therapies based on nanoparticles from therapies employing small drug molecules, and lead to an alteration of the therapeutic concept. In principle, the impossibility of particles entering a non-target tissue underlies the potential of creating therapies without side-effects. To exert therapeutic action, the particles must cross those barriers which hide the lesion, however. Since the particles cannot enter a tissue by exploiting concentration gradients, they must either evade the blood-tissue barriers or be ferried across them. The barriers can be evaded by (1) opening them, by (2) exploiting EPR, or by (3) evading them by utilising an entirely different application route, such as for example using the olfactory pathway to evade the blood-brain barrier. Finally, (4) the invasive capacities of the immune system can be harnessed, and since immune cells can be addressed within the bloodstream, such immune therapy avoids the necessity of confronting blood-tissue barriers. These four options are discussed below. However, in a large number of clinically important cases, evasion is not possible (see below). In order to provide therapy in these cases, nanoparticles must be designed which can penetrate *intact* vascular barriers and target lesions behind *intact* blood-tissue barriers. This review uses the terminology “transbarrier targeting” to describe such passage of nanoparticles through *intact* vascular barriers, with subsequent accumulation of the nanoparticles at target molecules within a lesion. Transbarrier targeting can be visualised in terms of “*flags and ferrying”* (Figure 5). Characteristic, specific molecules displayed on the target endothelial cell apical surface serve as *flags*, revealing that a lesion hides behind this patch of endothelium, thus “*flagging*” the lesion and allowing particles to “recognise” that they should leave the bloodstream here and cross the endothelial cell layer at this site. The nanoparticles must be equipped in order to recognise that this patch of endothelial surface is flagged. They must also be equipped to achieve transendothelial passage at this site (and nowhere else), in order to access the underlying perivascular space there. The nanoparticles must bind specifically to the flagged target endothelium. After binding to the “*flag*” molecules identifying the target endothelium, they must next co-opt the transport mechanisms available there and use them as “*ferrying*” systems to cross into the hidden lesion. Such transport mechanisms are available at the luminal side of every blood-tissue barrier. Each step of this “*flag and ferry*” approach has already been shown to be feasible (see below).

### 3.3. Evading Blood-Tissue Barriers

The four possible ways to evade blood-tissue barriers were noted above. These are by opening the barrier, by exploiting EPR, by selecting a route to the target which avoids confronting a particular barrier, and by exploiting the capabilities of the immune system. These four possibilities are discussed next.

#### 3.3.1. Opening the barrier

**Figure 6:**
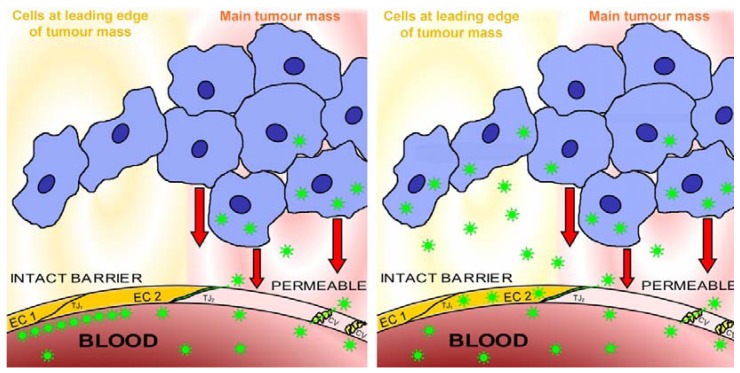
Transbarrier targeting depends on nanoparticles passing through an intact vascular barrier. The image at left shows the present state of the art in nanomedicine. Nanoparticles are prevented from accessing the lesion by endothelial cells maintaining intact barriers, so that no nanoparticles (shown as green stars) penetrate into the perivascular compartment (pale yellow) beyond the barrier-forming endothelial cells EC1 and EC2 (bright yellow to represent barrier function, resulting partly from closed tight junctions such as TJ_1_). The perivascular compartment is accessed by nanoparticles only at sites (pink) where the endothelial cell layer is permeable (pale pink to represent absence of barrier function, due to open tight junctions such as TJ_2_ and to caveolae (cv)), for example where vessels respond to factors such as HIF-α (represented by red arrows), secreted by hypoxic tumours (shown as a mass of blue cells) into their environment. The image at right shows transbarrier passage of the nanoparticles, seen as green stars crossing the barrier-maintaining endothelial cells EC1 and EC2 (yellow). Note that these endothelial cells do not become permeable, but continue to maintain the barrier while the nanoparticles cross them. Once past the intact blood-tissue barrier maintained by the non-permeable endothelial cells, the nanoparticles enter the interstitium behind the barrier, the protected state of this interstitium is denoted by yellow shading. Behind the barrier, in the protected interstitium, the nanoparticles can access a narrow strand of tumour cells (blue) which are well-oxygenated and release no factors (compare Figure 4). These cells, left alive, would generate metastases, but after being accessed by the nanoparticles they can be destroyed by the drug which the nanoparticles release.

The first way of evading the barrier is to eliminate it by chemical treatment. Nanoparticles can enter the perivascular spaces (Figure 6) when vascular barriers are opened by application of an osmotically active agent such as arachidonic acid, the eicosanoids, bradykinin, histamine, free radicals, ormannitol [187], by inflammatory processes [188], or by the vascular response to factors produced by tumours in response to hypoxia [189] (Figure 4). Detergents have also been used to coat nanoparticles to aid passage through the blood-brain barrier [190,191].

#### 3.3.2. EPR

The second way to avoid a blood-tissue barrier is utilise pathological conditions in which the barrier has been eliminated as part of the disease process. In the present state of the art, nanoparticles are typically targeted to cells and molecules readily accessible in the blood (e.g.: fibrin) [192,193] or located behind permeable endothelial sheets (e.g.: in advanced metastatic tumours) [194]. A wide range of nanoparticles has been shown to pass through damaged barriers, particularly byEPR [195,196,197]. EPR is the basis of delivery of “biological” drugs (mainly monoclonal antibodies) at present, for example Abraxane^®^ [194]. This allows access to large masses of hypoxic tumour tissues which produce factors such as HIF-α, which render endothelial cells permeable; it does not provide access to small numbers of tumour cells hidden by blood-tissue barriers (see above) (Figure 4); failure to eradicate these latter cells implies a high probability of metastasis at a later time. Tumour cells in micrometastases also are not eradicated, since they also hide behind blood-tissue barriers. Exploiting EPR is only a partially successfully strategy, because it cannot lead to eradication of all the tumour cells.

#### 3.3.3. Olfactory route to evade the blood-brain barrier

**Figure 7:**
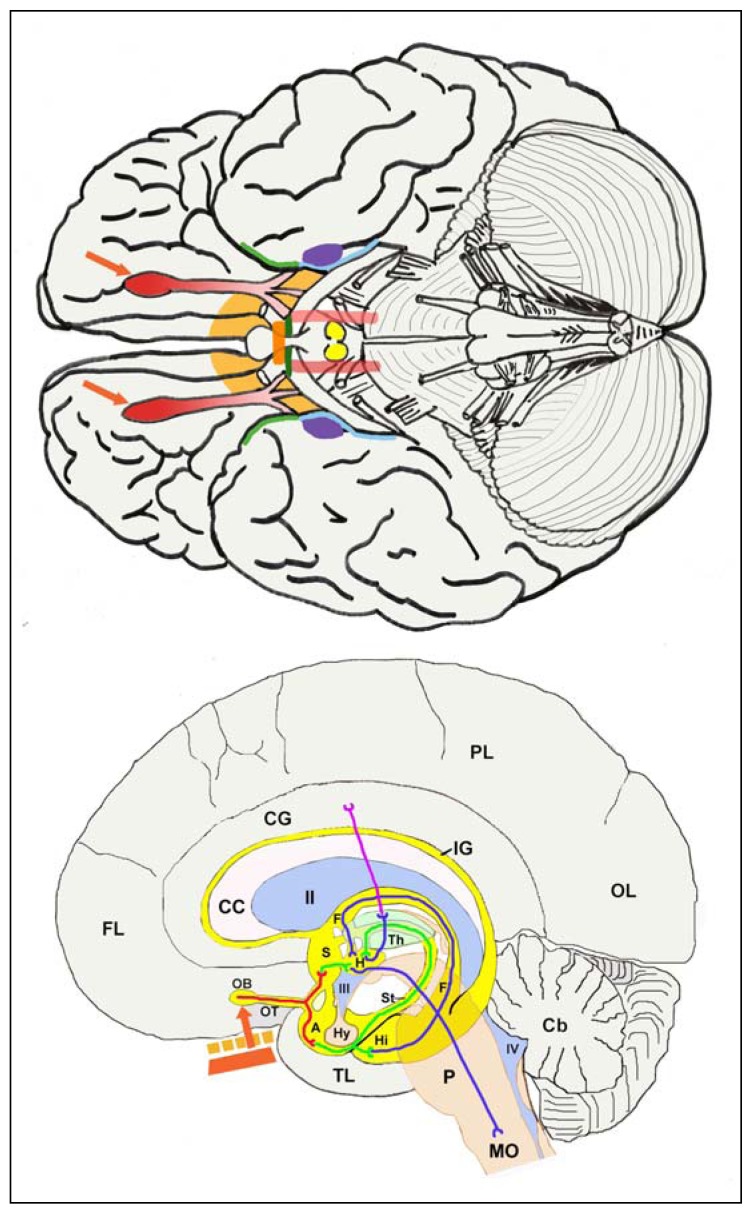
The Figure shows the olfactory pathway connections as seen from the ventral surface of the brain (upper image) and as projected onto a sagittal view of the brain (lower image). *The upper image:* a view of the brain commonly presented in neuroanatomical textbooks, is colour coded to show the major destinations and some of the pathways of the olfactory system. The colours are coded as follows: Red: the two olfactory bulbs, continuous with the two olfactory tracts shown as red fading to pink; Orange-yellow: the anterior perforated substance and the septal area; Bright orange bar: the anterior commissure; Green: pre-piriform cortex; Pale blue: entorhinal cortex; Purple: amygdala; Pale red bars: medial forebrain bundles; Yellow: mammillary bodies; Grey: the major mass of the brain which has little or no direct connection with the olfactory pathways; red arrows: inputs from the olfactory epithelia to the olfactory bulbs. It is evident from this image that the olfactory system is double, being present in both halves of the brain. It is also evident that the majority of the brain volume cannot be reached via these pathways. *The lower image:* a sagittal view in which several parasagittal planes and also the sagittal plane are superimposed to provide a composite sagittal image in which the major structures associated with the olfactory pathways are shown in relation to the whole brain. The olfactory pathways, shown here bright yellow, are shown single in this image, but reference to the upper image of this Figure will show that the pathways are present twice, one in each half of the brain. The structures associated with the olfactory pathways, and also the fibres running in those pathways, are colour-coded in this image. The coding is as follows: Grey: the major mass of the brain which has little or no direct connection with the olfactory pathways; Bright Yellow: the olfactory pathways; Pale Pink: the corpus callosum; Pale Brown: the midline structures of the brain; Blue: the ventricle system of the brain; Pale green: the thalamus; Red bar: the olfactory epithelium; Orange bar with white gaps: the cribriform plate of the ethmoid bone. The labels showing the brain structures are coded as follows: the cerebral cortex is labelled with FL (frontal cortex), PL (parietal cortex), OL (occipital cortex), TL (temporal cortex). The olfactory bulb (OB), receiving sensory inputs from the olfactory epithelium (red bar) connects via the olfactory tract (OT) to the olfactory pathways. The cingulate gyrus of the cortex is labelled CG. Cb: cerebellum. IG: indusium griseum; CC: corpus callosum; S: septal nuclei; A: amgydala; Hy: hypophysis; Hi: hippocampus; P: pons; MO: medulla oblongata; St: stria terminalis; Th: thalamus; F: fornix; II: the two lateral ventricles; III: third ventricle; IV: fourth ventricle. The nerve fibres running in the olfactory pathways are also colour-coded: the numerous olfactory epithelial cell fibres are shown as a single red block arrow; the neurites passing from the first synapse, the mitral synapse in the olfactory bulb, are also shown red; neurites from secondary neurons are shown green, from third-order connections are shown dark blue, and fourth-order connections are purple. Note that at each of the synapses in the chain, the proportion of nanoparticles that can pass through is small, so that the serial dilution of the original dose is very large. Note also that the olfactory pathways reside primarily in the central core of the brain, and that the (large!) volumes of the cerebral cortex and the cerebellum, and also several regions of the midbrain, receive essentially no inputs from the olfactory pathways.

The third way to evade a blood-tissue barrier is to apply the drug via a different route which accesses the same tissue indirectly. An interesting example has been the attempt to evade the blood-brain barrier in this way. Several research groups have considered targeting the CNS tissues via nasal application of nanoparticles, that is, via uptake into the olfactory neurons which reside in, and have sensory endings in, the olfactory mucosal epithelium [198]. We digress here to examine whether the blood-brain barrier could be avoided by intranasal application of nanoparticles. The olfactory sensory cells project to the olfactory bulb and make synapses onto the mitral cells in the synaptic glomeruli there [199]; to enter these second-order neurons, nanoparticles must cross the synaptic cleft. Nanoparticles can indeed pass synaptic clefts when previously applied to the neuronal membrane surface [200], or when loaded into synaptic vesicles [201]. Furthermore, nanoparticles loaded into endocytotic vesicles can be transported in both the anterograde [202] and retrograde [202,203] directions. At first glance, it appears that intranasally applied nanoparticles can reach the brain. However, only a small fraction of nanoparticles applied to the olfactory epithelium would enter the retrograde transport to the olfactory bulb, and an even smaller fraction would cross to the mitral cells and enter the anterograde transport in the secondary projection forming the olfactory tract to the deeper-lying brain regions. In addition to these quantitative considerations, toxicological aspects must be considered [204], concerning the extent to which the nanoparticles disturb normal neuronal functioning. Having entered the secondary projection fibers, the nanoparticles could be transported to several sites via anterograde transport: in the medial olfactory tract to the anterior perforated substance and paraolfactory (septal) area, or via the anterior commissure to the contralateral septal area; in the lateral olfactory tract they could be transported to the prepiriform area of the temporal lobe of the(3-layered) allocortex, to the amygdaloid nucleus. They could be transported to the secondary olfactory cortex (the entorhinal area of the parahippocampal gyrus - of some potential clinical interest because this area of the cortex exhibits alterations at one of the earliest stages of Alzheimer’s Disease [205]), to the lateral preoptic area, the amygdaloid nucleus and via the medial forebrain bundle to the brainstem. They could also reach the hypothalamus via direct connections from the septal area, or via the stria terminalis and fornix, and from the hypothalamus to the brainstem via the dorsal longitudinal fasciculus; finally they could also reach the thalamus via the mamillothalamic tract, and from the thalamus they could reach the cingulate gyrus. Figure 7 maps the most important of these routes; it also shows that passage to each of these destinations involves multi-synapse crossings and anterograde transport along chains of 3-4 axons. Each of these crossings sharply reduces the rate and number of nanoparticles transferred. In principle, the nanoparticles would indeed reach all these destinations, but they would do so in extremely diluted amounts. The olfactory bulbs are directly connected with vegetative and integrative areas localized in the hypothalamus and the brainstem and with the major aminergic nuclei that play an essential role in the neurovegetative, neuroendocrine and behavioral regulations. However, the major bulk of the brain tissues cannot be reached via pathways originating in the olfactory system, including most of the diencephalon and telencephalon, almost all of the mesencephalon, almost all of the metencephalon, and almost all of the myelencephalon. This strongly limits the disease entities which can (in principle) be accessed in the CNS from the nasal epithelium. More important as a practical consideration is that all oncological disease states in the central nervous tissues are much more accessible via the EPR effect.

#### 3.3.4. Immune therapy

The fourth way to evade a blood-tissue barrier is to utilise the capacity of certain cells to cross the barrier during the course of normal tissue surveillance. The most important example is immunotherapy, which targets cells present in the blood, aiming to alter the behaviour of the immune system and thus lead to immune attack upon the tumour. The success of this approach is reflected in enhanced overall therapeutic efficiency; due to amplification (via cellular cascades) occuring within the immune system [206,207,208].

### 3.4. Confronting Intact Blood-Tissue Barriers: Penetrating Barriers by Flagging and Ferrying

#### 3.4.1. Flagging

Flagging is familiar in the form of selectin expression on activated endothelial cells in high endothelial venules in inflamed tissues [209,210,211,212]; atherosclerotic lesions are also flagged by adhesion molecules on overlying endothelial cell surfaces [213,214]; proliferative and tumour-induced “markers” for microvascular endothelium within tumours provide a further form of vessel flagging [215,216,217,218,219]. In all these cases, the expression of a specific molecule at the endothelial cell apical surface “flags” the presence of a lesion in the perivascular space behind the microvessel wall. It may not always be necessary to exploit the presence of the flag molecules: the microvessel may be permeable at this site, due to the action of factors released by the lesion, and in this case the EPR effect can be exploited to attack the lesion. In other cases the flag molecules are present on non-permeable endothelial cells: this is much more important, because the lesion hiding behind non-permeable sites of the microvessel wall cannot be accessed via EPR. In such cases the nanoparticles must be ferried across the endothelial cell layer. In the same way that leucocytes “roll” on selectin-flagged endothelial cell surfaces, and finally come to rest there, attached by tethers and strong bonds to the endothelial surface (see below), nanoparticles bearing targeting groups directed towards the flag molecules will attach to the flag molecules and thus become attached to the target endothelial cell apical surface. This has been demonstrated to occur for nanoparticles targeted to selectins [220]. The bound particles can now exit the bloodstream by crossing the endothelial cell layer to enter the underlying perivascular space (or “interstitium”). Lesion-flagging molecules are obviously of great value, and as an essential step in developing Nanomedicine, organ-specific and/or lesion-specific flag molecules will be identified for many tissues and lesions, a process of discovery which is already well under way. Not only have suitable flag molecules already been identified, e.g., for the lung [186], but methods of searching for them systematically have been described. These include proteomic screening techniques [221] and phage library screening techniques [222].

#### 3.4.2. Ferrying

At blood-tissue barriers the paracellular path is closed to nanoparticles. In order to access the underlying perivascular space, the particles must be transported, *“ferried”*, across the endothelial cell layer. For “natural” nanoparticles, typically macromolecules and usually proteins, such as transferrin [223,224,225,226], albumin [227], cationized albumin [228], or Apo-E-coated proteins [229,230], transport mechanisms are present in the endothelial cell to facilitate their movement out of the blood, across the cells, and into the interstitium. These natural transport mechanisms can be exploited by nanoparticles made of materials such as transferrin, for which appropriate receptors exist on the surfaces of the endothelial cells at barriers [224,225]; a further alternative is to attach ligands for such receptors to nanoparticles made of any other material, for example high density lipoprotein (HDL) [231,232,233]. In this way, the natural transport mechanisms for selected molecules can be “hijacked” for therapeutic purposes. Exploitation of these ubiquitous pathways (transferrin, albumin) will however generally ferry the particles into many tissues, including numerous sites in addition to the lesion. For therapeutic purposes it is more effective to exploit transport mechanisms closely associated with flag molecules. In this way, flagging and ferrying can be combined in a single recognition and transport system.

Two different vesicular systems allowing transendothelial transport could in principle be used. A third system, pinocytosis, takes the pinocytosed material into secondary lysosomes and does not cross the cell [234], so is not useful for therapeutic purposes. The two ferry systems which are of interest for transporting nanoparticles are therefore transcytosis in clathrin-coated and other endocytotic vesicles, and transcytosis in caveolae [235,236,237].

a. Receptor-mediated transcytosis has long been associated with clathrin-coated vesicles [238,239,240,241]. A long-running controversy concerning the existence and importance of vesicular transendothelial transport has been partly settled in favour of the major energy-dependent form of transport involving caveolae [242]. Endothelial cells are not the only cells capable of receptor-mediated transport. An interesting case is found in the central nervous system, where astrocytes possess receptors for serum albumin [243], and both in intact brains [244,245] and in culture conditions [227] can take up albumin by receptor-mediated endocytosis, and pass it through the cells by transcytosis [246,247]. They can transcytose 10 nm gold particles coated with serum albumin [227], and such particles have a total diameter 16-20 nm because albumin is 3 × 8 nm in size [248,249]. At the blood-brain barrier, therefore, albumin can be transported first through the endothelial cells and then later through the astrocytes, in a sequential transport which carries the protein (and, in principle, particles) from the bloodstream through into the interstitial space surrounding the neurons.

b. Caveolae. These are flask-shaped invaginations at endothelial cell luminal surfaces, >70 nm in diameter [250,251,252], which bud from the cell membrane [253,254] and deliver their contents to the Golgi system [255,256,257,258,259], to the endoplasmic reticulum [258,260,261,262] or to lysosomes [263,264], and in endothelial cells can transcytose their contents across the cell into the underlying perivascularspace [18,186,250,265,266]. The endothelial cell caveolae can be targeted organ-specifically by monoclonal antibodies, for example for lung, and can transport ~ 90% of the initial dose to the lung within 30 minutes [186]. Sequential transport, first through the alveolar endothelial cells and then through the underlying epithelial cells, has been demonstrated also for caveolae [186].

It has therefore been shown that appropriately targeted particles can access lesions by flag-and-ferry mechanisms, and that the efficiency of such specific transbarrier targeting can be very high. The uptake efficiency into lung tissue reported by McIntosh *et al.* [186] and Carver and Schnitzer [250] is as high as 89% for targeted gold nanoparticles, whereas 200-fold less of control non-targeted nanoparticles entered the lung and remained in the blood. Application of nanoparticles, which cannot cross barriers unless designed to do so, therefore allows drug delivery rates to be achieved which are more than one hundred times larger than those typically achieved by use of small drug molecules, and without simultaneous delivery to numerous non-target organs. It remains to translate these principles into clinical and commercial reality.

## 4. Design of Barrier-Passing Nanoparticles

The design requirements are those commonly recognised: to obtain maximal therapeutical efficiencies, and to minimise side-effects, to ensure the particles do not release drug molecules in non-target tissues, do not cause toxicity or immunogenicity, and - if they fail to target - are cleared rapidly from the organism. These principles can be realised by use of nanoparticles designed for transbarrier targeting. Nanoparticles do not cross intact blood-tissue barriers unless designed to do so. They must be ferried across the barriers, and rational design will equip them with the apparatus necessary to achieve this. The rewards will be high: in principle, drug delivery could be enhanced from its present typical efficiency of much less than 1% to a rate close to 100%. The apparatus which must be designed into the nanoparticles includes the targeting apparatus, which will be considered next. The multistage nature of the targeting path from bloodstream to lesion requires particles to pass at least two, usually three, barriers to arrive at the lesion cell. It is therefore useful to be familiar with the properties of natural inanimate particles that also target particular cells and tissues, and viruses provide a useful model, to which we will refer for guidance.

### 4.1. Transbarrier Targeting as Essential Requirement

Double targeting is a minimum requirement for nanoparticles which must navigate a multistage pathway. They will encounter a first set of target molecules on the target endothelium, see *stage 2* above, and later they will encounter a second set of target molecules on the target lesion cells, see *stage 4* above. For nanoparticles directed towards intracellular or even intranuclear targets such as DNA, after they have passed the four targeting stages discussed in section 2 above and have encountered one set of targeting molecules on the microvascular endothelium and a second set on the cell membrane of the lesion cell, they will next possibly encounter a third set at the nuclear membrane of the lesion cells and finally a fourth set of target molecules within the lesion cell nucleus; this review does not follow the particles or drugs into the cells to their intracellular targets, because this topic has recently been well reviewed by Torchilin [40].

### 4.2. The Numbers of Targeting Groups per Nanoparticle

**Figure 8:**
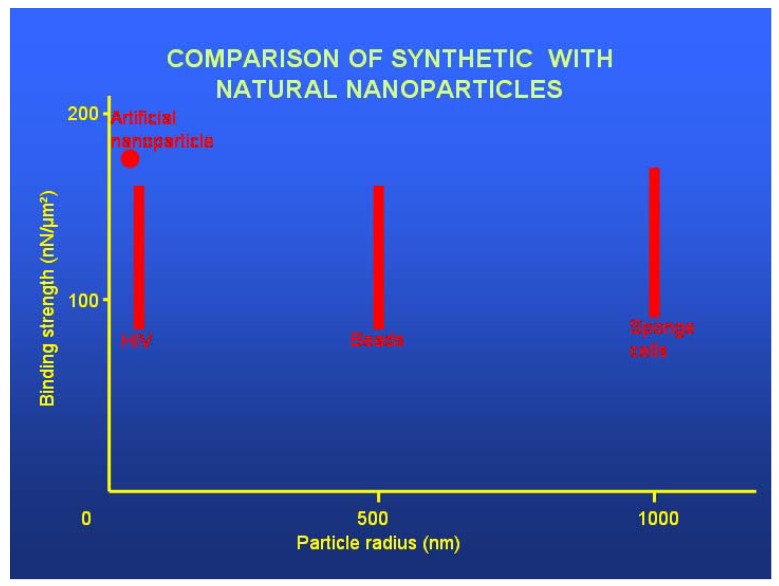
The density and strength of molecule pair interactions is invariant across the nanometer-micrometer size range from 20-100 nm diameter as shown by the artificial nanoparticles and the HIV virion, to approximately 1 micrometer, as shown by the sponge cells: the magnitude of the interactions is constant as shown by the horizontal pattern formed by the interaction strengths.

The properties of targeted natural nanoparticles offer a guideline, for example the viruses. The wild-type human immunodeficiency virus HIV-1 bears 8-10 trimer targeting spikes and has a diameter ~126 nm [267], which corresponds to an average spacing of one trimer targeting spike for each 5,000 nm^2^ of viral surface area. The trimer targeting spike bears three targeting molecules, each capable of entering into a molecular binding interaction with a specific molecule on the target cell surface [268,269]. Since a 30 nm diameter nanoparticle has a surface area of only about 1,000 nm^2^, 1-3 correctly oriented targeting “spikes” should suffice for specific binding of a nanoparticle of this size to its target molecule.Zhu *et al.* [267] report that the HIV-1 targeting spikes are 8 nm long and spaced approximately 16 nm apart, which provides a conceptual framework for considering the distance required between the nanoparticle surface and the stereospecific binding groups. The targeting spike of the virion, like the microvillus of the mammalian neutrophil [270], extends the specific interacting molecules, which mediate binding and attachment, towards their partners on the target cell. These interacting molecular pairs, once they come within a few tens of nanometers of one another, bind to one another with a binding force which has been measured directly in several different ways. In addition to the geometry of the interaction, the strength of the binding force is one of the major parameters involved in specific targeting. Specific binding involved in recognition events occurs by means of forces that are non-covalent, and reversible, and are on the scale of a few hundred picoNewtons (pN). The binding force on an antibody molecule has been reported as ~ s120 pN [271], or as ~ 244 pN, with a binding lifetime of a fewseconds [272,273]. The binding force for a protein-glycan molecular interaction is 165 pN [274,275]. Carbohydrate-carbohydrate specific interactions commonly found in biological recognition systems [52] exert binding forces between identical glycans of ~250 pN and between heterogenous proteoglycan molecules of 50-400 pN [276,277]. Translating these forces into the force required to tether cells, the minimum force required for formation of a neutrophil membrane tether is 45 pN [278], and similar forces are required to pull tethers from erythrocytes [279] and neuronal growth cones [280,281]; in these cases, calculation shows that at the point of molecular attachment of the tether the force is 112 pN, with a bond lifetime of 0.29 seconds [282]. All these forces of a few hundred pN, as required for attachment via specific molecule interactions, are about 100 × too small to disrupt cell membranes, which failat ~ 10^4^ pN/µm [278]. For interactions between bulky structures such as cells, attachments via single molecule pairs are not sufficient, and multiple molecular interactions mediate the tethering, attachment and aggregations of the cells: in living sponge cells (diameter 2.0 µm), cell aggregation requires 828 glycan interactions/µm^2^and a similar number (796 glycan interactions/µm^2^) is required to aggregate 1.0 µm diameter beads [52]. The HIV-1 wild-type virion, 130 nm in diameter and bearing 8-10 trimer targeting spikes, has a spike density equivalent to 170 trimer spikes/µm^2^, mediating510 interactions/µm^2^. Note that the sponge cell has a volume of 4.2 µm^3^, the bead a volume of 0.5 µm^3^, the neutrophil (radius 4.25 µm) a volume of 321 µm^3^ and the HIV virion a volume of 0.0012 µm^3^, then the volume relationships are in the following proportions: HIV-1 virion = 1, bead = 417, sponge cell = 3,500, neutrophil = 267,500. The density and strength of molecule pair interactions therefore appears to be invariant across the nanometer-micrometer size range (Figure 8). The neutrophil does not slow its large bulk, travelling at high speed in the bloodstream, by forming more and stronger bonds, but rather by use of special “slipping and catching” bonds, and by extending microvilli (with the binding molecules at their tips) to act as springs [270]. In sum, consideration of the parameters found in the wide range of specific targeting attachments found in nature suggests that a molecular interaction should be chosen which has a binding strength 50-300 pN, and that the binding molecule on the nanoparticle should be located on a stalk or spike, which for nanoparticles of diameter 30 nm should be 2-5 nm long; one or two such stalks, giving a binding site density ~ 500/µm^2^, should be sufficient to anchor the nanoparticle to a cell surface in a physiologically “normal” manner.

### 4.3. Targeting Chemistry

The discussion so far has focussed on targeting the nanoparticles towards specific characteristic molecules whose presence indicates the existence or proximity of a lesion. This type of targeting can be considered as positive targeting, which directs the targeted particle towards the lesion. As reviewed earlier [180], a wide range of molecule types capable of high-affinity stereospecific binding is available for use of targeting groups, these include antibodies, aptamers, enzymes, lectins, nucleotides. Any of these molecules can be attached to a nanoparticle to ensure its binding to the target molecule, once the targeted particle has come within a distance of approximately 50 nm of the target molecule. These targeting molecules are essential in attachment of the particle to the flag molecules on the target endothelium in the microvessel within the lesion, and again in attachment of the particle to the flag molecules on the surface of the lesion cells. By use of antibody molecules, McIntosh *et al.* [186] demonstrated drug delivery rates of approximately 90% by use of gold nanoparticles. In cases where positive targeting is not enough - and at this stage of development in Nanomedicine there is too little experience to state what these circumstances may be - the particles need to carry a further type of molecule which prevents their attachment to non-target sites, or their uptake into cells of the body’s surveillance system. This type of molecule can be considered a form of negative targeting, and has become familiar amongst researchers working with nanoparticles as conferring “stealth” properties on the particles. By modifying the particle surface additionally with appropriate electric charges, with hydrophilic groups, or by addition of PEG-chains [283], particles can be made less likely to be taken up into elements of the reticulo-endothelial system such as macrophages, less likely to enter the liver, and more likely to undergo renal clearance. These features have been well reviewed [284,285,286]. However, some aspects have been less frequently considered, and amongst these the potential immunogenicity of the particles is a prime example. Nanoparticles can be constructed which function as excellent adjuvants in immunisation protocols [287,288,289,290], and it is likely that the size and surface characteristics of nanoparticles confer on all nanoparticles more-or-less potent adjuvant properties. For these reasons, the immunogenicity of the particles should be reduced to the minimum. One technique for doing this is to ensure that all glycosylated components of the particles are humanised [291,292,293,294].

### 4.4. Nanoparticle Chemistry

There are numerous types and chemistries of nanoparticles [185]. Some earlier and also recent significant research on targeting of nanoparticles was carried out by use of gold nanoparticles, for example by the groups around Palade [251] and Schnitzer [186]. Gold particles are unreactive in the physiology of the human body, so do not exhibit the interactions with body fluids and cells that complicate research with some other types of nanoparticle. However, they are not well adapted for clinical diagnostics *in vivo* or for controlled release of drugs; they have provided an excellent test-bed for development of innovative targeting modalities, and will continue to do so in the future. For clinical applications, a wide range of particles prepared on an organic basis offers a more useful approach to drug delivery. First amongst these one might consider to be particles prepared on the basis of the body’s transport protein, albumin [295,296], which has been demonstrated to transport and release essentially all drug types [297,298]. Polymer-based syntheses also offer immense flexibility in drug transport and release [299,300], as do dendrimer-based particles [301,302,303]. Generally, the most widely studied nanoparticle type has been the liposome [28,173,195].

### 4.5. Nanoparticle Size

Selecting an appropriate size of the particles aids in selecting a particular pharmacokinetic modus, thus particles larger than 150 nm diameter will be engulfed by macrophages, those 60-150 nm diameter will cross cells in caveolae, and those 20-60 nm diameter can cross cells by transcytosis in coated vesicles, or enter the endosomal-lysosomal pathway. As noted above, nanoparticle size apparently implies immunological capacities as adjuvant, a clinically significant relationship which requires further study. Furthermore, the drug dose which can be packed into a nanoparticle depends both on the packing density and the size of the particle. The size of the nanoparticles should be standardised, because in the nanoscale size range surface area varies strongly with particle diameter, so that the small diameter difference between 15 nm and 25 nm translates into a surface area difference which requires more than three hundred human bodies (each having a surface area close to 1.5 m^2^) to equal it [296]. It is therefore essential that the nanoparticle size dispersity, measured as polydispersity index (PDI), should be low, best < 1.1. Furthermore, in addition to carrying different amounts of drugs, particles of different sizes will be subjected to different *in vivo* pharmacokinetics (see above). During synthesis, size selection by repeated filtration should be avoided, because filtration stresses and disrupts the particles.

### 4.6. Nanoparticle Stability and Flexibility

In the bloodstream, the particles encounter mechanical stress; the blood flow in a human adult is ~ 5-30 liters per second through the major vessels (depending on rest or exercise). Bloodstream turbulences tend to disrupt particles, and passage through cells comprising the tissue barriers, with their different microenvironments, also have significant potential to break up the particles and can best be resisted if the particles have thixotropic (flexible) characteristics. Enzymes (esterases, proteolytic enzymes, and others) present in the blood and in cellular compartments attack the chemical integrity of organic-based particles. These disruptive influences can also be used to design particles with pre-programmed failure points, e.g., amide-linkages are cut by amidohydrolases and are more stable than ester-linkages which are cut by esterases. Building such failure points into the structure of a nanoparticle allows the inclusion of small, well-defined nanoparticle components in which nanoparticle breakdown characteristics can be defined by the components' size, hydrophobicity or hydrophilicity. This allows nanoparticle breakdown modus and timing to be pre-programmed, and also the nature of the breakdown products to be pre-selected. In principle it allows nanoparticles to be designed which persist in the blood for a predetermined number of target molecule turnover cycles (see Section 4.7, below).

### 4.7. Control of Nanoparticle Clearance

Nanoparticles must accumulate in the target lesion. This requires binding of the particles to certain sets of target molecules in cell membranes, as discussed above (see Section 2). The target molecules are rapidly saturated, and their turnover time is typically about 20 minutes: most nanoparticles will not achieve target molecule binding during their first pass through the circulation. However, each target molecule can be used several times, and will bind blood-borne nanoparticles rapidly once returned to the cell surface. As a result, many nanoparticles will remain in the bloodstream for longer than three hours before they can bind to their specific target molecules (counting several turnover times of the target molecules). During this time, these nanoparticles should remain in the blood and not be cleared from it by the macrophages of the reticuloendothelial system. The particles should therefore be designed to evade uptake into the macrophages of the reticuloendothelial system, predominantly the macrophages of the liver, spleen, bone marrow and lymphoid organs [304,305]. To achieve such design, the clearance mechanisms need to be understood. Studies of clearance mechanisms have been mostly carried out using liposomes [304,305,306,307,308]. We expect that principles learned from the study of liposomes will be largely applicable to other types of nanoparticles also. Phagocytosis of the particles follows coating of the particles with “opsonins”, proteins such as the immunoglobulins IgG1 and IgG3, or components of the complement system (C3b, iC3b, C1q), and others including fibronectin and lipopolysaccharide-binding protein. When liposomes are introduced into the bloodstream, opsonins adhere to them in patterns and amounts depending on the physicochemical properties of theliposomes [309]. Later, by forming bridges between the particles and the phagocytes, the opsonins promote phagocytosis [310]. The detailed mechanisms vary between phagocytosis in the liver and in the spleen or bone marrow [304,311]; there are also species differences [304,312]. Phagocytic uptake can be evaded by preventing opsonisation of the particles. This can be carried out by attaching molecules such as linear and branched polyethylene glycol (PEG) [313,314], polyacrylamide, polyvinylpyrrolidone, polyacryloyl morpholine [313] or dextrans [315] to the particles. Finally, for nanoparticles not reaching the target after numerous passes through the circulation, a pre-programmed mode of breakdown can be incorporated into the chemistry of the particles by inclusion of appropriate chemical linkages as described above (*section 4.6*).

### 4.8. Market and Regulatory Issues

Like any product designed for pharmaceutical application, targeted nanoparticles are subject to overview by regulatory authorities, so that their synthesis and testing must conform to guidelines and laws. These apply primarily to toxicological properties and to animal testing of the products.

*1. Non-toxic nanoparticle educts*: As occurs with any substance entering the living body, no matter whether medication or food, nanoparticles will be subjected to metabolic reactions within the body. Even though they can be disguised chemically (see above) they will encounter some degradation on their way to the target, inside the circulation, when crossing the blood-tissue barriers and especially when they fail to reach the target at all. It is therefore essential that the single components used for the nanoparticle synthesis are non-toxic, that the whole nanoparticulate system does not cause immunogenic or even toxic reactions *in vivo* and therefore includes a pre-programmed breakdown mechanism. Planned breakdown can be achieved by inclusion of covalent bonds having different *in vivo* stabilities. A crucial matter here represents the purification method chosen; simple dialysis often results in poorly cleaned products leaving remnant educts in the sample which further disturb any pharmacokinetic evaluation [296]. Dialysis is often better replaced by diafiltration or size exclusion chromatography. It should be mentioned here that no regulatory guidelines have been set up for *ex vivo* and *in vivo* immunological as well as toxicological studies, with the exception of the OECD guidelines [316]; regulatory uncertainty complicates industrial production and clinical application.

*2. GMP production*: In designing nanoparticulate systems for *in vivo* medical application an essential requirement is the sterilisation of the product. Sterilisation can be conducted in different ways (by heating, by irradiation, by filtration). Since organic-based nanoparticles normally are heat-labile, sterilisation methods using heat should be excluded. Sterile filtration excludes bacteria >0.2 µm diameter but allows e.g. *Mycoplasmas* and *Spirochaetes* or viruses to pass the filter. Furthermore, nanoparticles >200 nm, which includes most liposomal systems, are destroyed by such filtration. As nanoparticles tend to aggregate during storage, proper storage conditions are required, including chemical and/or physical modifications (*i.e.*: coating with oligosaccharides, coating with charged phospholipids), which may involve logistical challenges.

## 5. Future Directions

Nanoparticles designed for transbarrier targeting, and exhibiting targeting efficiency of ~90%, will have numerous uses in oncology, in transplantation surgery, in therapy of atheroses, in therapy of diabetes and other autoimmune diseases, in treatment of psychoses and of dementias. Successful clinical application in these areas would facilitate personalised medicine. The topic of this review is therefore of prime importance for the future of Nanomedicine. To succeed in establishing a new paradigm of drug delivery, nanomedicine must understand the blood-tissue barriers, which form the foundation for its potential specificity and high efficiency. In order to obtain a clear understanding of the particular blood-tissue barrier in any particular organ, several types of evidence will be required, and data should be collected from the various developmental and functional phases of that particular organ. At present, only one type of blood-tissue barrier - the blood-brain barrier - has been investigated in this depth of detail, and even in this case important developmental data are lacking. Our understanding of blood-tissue barriers at present, therefore, is scanty at best. Transbarrier-targeted particles will be required, which will require targeting with at least two different targeting groups per particle to allow multistage access to the lesion-specific molecules. Development of such particles, with their ancillary properties as noted above, might be considered a worthwhile grand challenge.
